# Identification of a Pyroptosis-Related Gene Signature for Predicting Overall Survival and Response to Immunotherapy in Hepatocellular Carcinoma

**DOI:** 10.3389/fgene.2021.789296

**Published:** 2021-12-03

**Authors:** Susu Zheng, Xiaoying Xie, Xinkun Guo, Yanfang Wu, Guobin Chen, Xiaochun Chen, Meixia Wang, Tongchun Xue, Boheng Zhang

**Affiliations:** ^1^ Department of Hepatic Oncology, Xiamen Branch, Zhongshan Hospital, Fudan University, Xiamen, China; ^2^ Key Laboratory for Carcinogenesis and Cancer Invasion, The Chinese Ministry of Education, Zhongshan Hospital and Shanghai Medical School, The Liver Cancer Institute, Fudan University, Shanghai, China; ^3^ Center for Evidence-based Medicine, Shanghai Medical School, Fudan University, Shanghai, China

**Keywords:** pyroptosis, hepatocellular carcinoma, prognosis, immune infiltrates, immune checkpoint inhibitors

## Abstract

Pyroptosis is a novel kind of cellular necrosis and shown to be involved in cancer progression. However, the diverse expression, prognosis and associations with immune status of pyroptosis-related genes in Hepatocellular carcinoma (HCC) have yet to be analyzed. Herein, the expression profiles and corresponding clinical characteristics of HCC samples were collected from the Cancer Genome Atlas (TCGA) and Gene Expression Omnibus (GEO) databases. Then a pyroptosis-related gene signature was built by applying the least absolute shrinkage and selection operator (LASSO) Cox regression model from the TCGA cohort, while the GEO datasets were applied for verification. Twenty-four pyroptosis-related genes were found to be differentially expressed between HCC and normal samples. A five pyroptosis-related gene signature (GSDME, CASP8, SCAF11, NOD2, CASP6) was constructed according to LASSO Cox regression model. Patients in the low-risk group had better survival rates than those in the high-risk group. The risk score was proved to be an independent prognostic factor for overall survival (OS). The risk score correlated with immune infiltrations and immunotherapy responses. GSEA indicated that endocytosis, ubiquitin mediated proteolysis and regulation of autophagy were enriched in the high-risk group, while drug metabolism cytochrome P450 and tryptophan metabolism were enriched in the low-risk group. In conclusion, our pyroptosis-related gene signature can be used for survival prediction and may also predict the response of immunotherapy.

## Introduction

Hepatocellular carcinoma (HCC) accounts for the majority type of primary liver cancer and is the sixth most common and fourth deadly malignancy globally (Bray et al., 2018). Liver resection or transplantation is still the first choice for radical cure, while only a small portion of patients fit the bill. As a matter of fact, more than 70% of the patients are already at a middle-to-late stage when first diagnosed. Despite the improvement of several new treatments such as immunotherapy and target therapy, the 5 years survival rate has remained less than 20% (Allemani et al., 2015; Katsanos et al., 2017). Thus, it is of great importance to develop novel therapeutic targets and reliable prognostic models for medical decision making.

Pyroptosis, a novel defined programmed cell death, is characterized by the cleavage of gasdermins and the subsequent cell membrane ruptures and necrotic cell death (Rogers et al., 2017; Shi et al., 2017). Pyroptosis was involved in innate immunity and initially found to play a crucial part in fighting against infection (Shao et al., 2021). However, increasing evidences have suggested that pyroptosis also takes part in cancer development (Fang et al., 2020; Tan et al., 2020). Deletion of NLRP3 (one of the key components of pyroptosis) in transgenic mice was found to be correlated with colon cancer development (Dupaul-Chicoine et al., 2010). NLRP3 inflammasome may also drive liver injury and fibrosis and may contribute to the development of liver cancer (Wree et al., 2018). More recently, the pyroptosis-related gene signatures for ovarian cancer and lung cancer has been proved to be prognostic (Lin et al., 2021; Ye et al., 2021). Interestingly, pyroptosis-induced inflammation was found to synergize with anti-PD1 therapy through triggering antitumour immunity (Wang et al., 2020). In HCC, pyroptosis was proved to be restrained in HCC tissues and cells, small molecule drugs such as berberine, euxanthone and miltirone may induce HCC cell death through triggering pyroptosis (Chu et al., 2016; Chen et al., 2018; Zhang et al., 2020a). However, the role of different pytoptosis-related genes in HCC has not been reported. Here, we explored the diverse expression, prognosis, associations with immune status and response to immunotherapy of pytoptosis-related genes in HCC.

## Materials and Methods

### Data Source and Pyroptosis-Related Genes Definition

RNA sequencing data (fragment per kilobase million, FPKM) of 374 HCC patients and their corresponding clinical features were collected from the TCGA database (https://portal.gdc.cancer.gov/repository). Besides, another 438 tumor samples from three cohorts were obtained from the Gene Expression Omnibus (GEO) database (https://www.ncbi.nlm.nih.gov/geo/), including 242 samples of GSE14520, 115 samples of GSE76427, and 81 samples of GSE54236. The “limma” and “sva” package of R software was used for data normalization between the TCGA and GEO datasets as previously described (Gautier et al., 2004; Leek et al., 2012). All the data used in the present study was collected from public databases. It was then confirmed that all written informed consent was obtained. This research was approved by the Ethics Committee of Xiamen Branch, Zhongshan Hospital. According to prior studies, 33 pyroptosis-related genes were obtained (Lin et al., 2021; Ye et al., 2021). However, the expression data of NLRP6 and PLCG1 were not found in the GEO datasets. Finally, 31 pyroptosis-related genes were adopted for further analysis (See Supplementary Table S1).

### Development and Validation of a Prognostic Pyroptosis-Related Gene Signature

Differentially expressed genes (DEGs) between non-tumor tissues and HCC tissues were identified by the “limma” R package with a false discovery rate (FDR) < 0.05. Then the Univariate Cox analysis was adopted to find out the prognostic related genes (*p* < 0.05). DEGs with prognostic value were further analyzed by the LASSO Cox regression model with “glmnet” R package (Simon et al., 2011). Finally, a five pyroptosis-related gene signature was conducted. The risk score of each patient was calculated according to the pyroptosis-related gene expression level and its regression coefficient. HCC patients from the TCGA cohort were then divided into low-risk and high-risk groups according to the median risk score. Survival analysis and predictive accuracy were conducted by the “survminer” and “timeROC” R package. According to the same median risk score from the TCGA cohort, the GEO cohort was also divided into low-risk and high-risk groups for further validation. Clinical characteristics of the TCGA cohort (age, gender, tumor grade, tumor stage) and GEO cohort (gender), as well as the risk score were collected for univariate and multivariable Cox regression models to identify the independent prognostic factor. The proportional hazard assumption test was also conducted.

### Functional Enrichment Analysis

Kyoto Encyclopedia of Genes and Genomes (KEGG) analyses were conducted by gene set enrichment analysis (GSEA) according to the DEGs between the high- and low-risk group.

### Tumor Microenvironment and Immunophenoscore (IPS) Analysis

Single-sample gene set enrichment analysis (ssGSEA) was applied to explore the relationship between the risk score and immune cells, as well as immune-related pathways. Immunophenogram of the Cancer Immunome database (TCIA, https://tcia.at/home) was used for evaluating response to immune checkpoint inhibitors (ICIs) (Charoentong et al., 2017).

## Results

### DEGs Between HCC Tissues and Non-tumor Tissues

**FIGURE 1:**
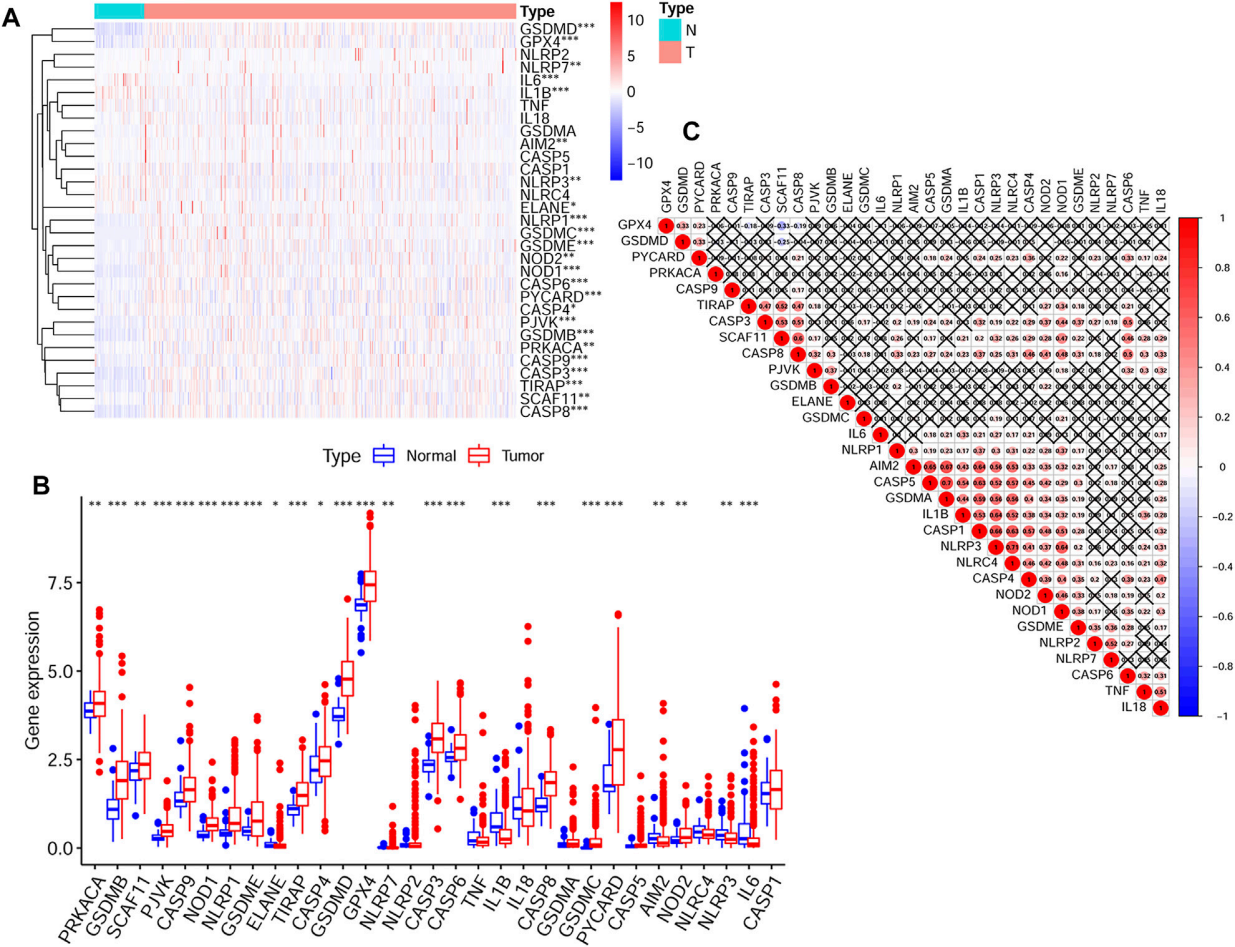
The transcription levels of 31 pyroptosis-related genes and the interactions among them **(A)** The transcription levels of 31 pyroptosis-related genes between tumor and normal tissues were shown in a heatmap **(B)** The transcription levels of 31 pyroptosis-related genes between tumor and normal tissues were shown in a box plot **(C)** The correlations between the pyroptosis-related genes (*p* values were represented as: ns not significant; *p < 0.05; **p < 0.01; ***p < 0.001.).

Expression data of 50 normal tissues and 374 HCC tissues were collected from the TCGA HCC cohort. And the expression of 31 pyroptosis-related genes were compared between these two groups. Finally, a total of 24 DEGs were obtained. The detailed information of selecting DEGs were shown in Supplementary Table S2. The expression levels and *p* values of difference between the 31 genes were shown in Figure 1A and Figure 1B. The correlations between these genes were shown in Figure 1C.

### HCC Subtypes Based on the DEGs

**FIGURE 2:**
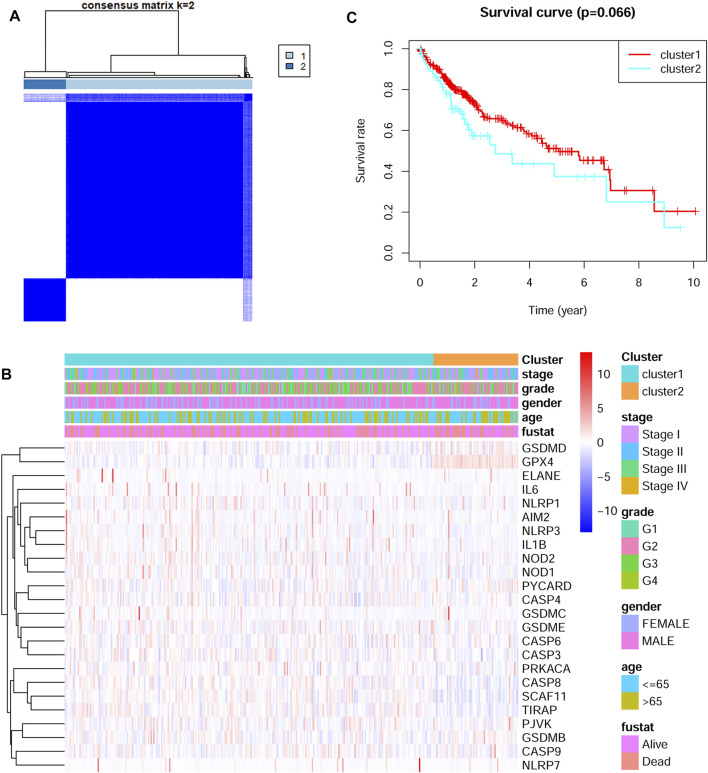
Identification of tumor subtypes based on the pyroptosis-related DEGs **(A)** Two clusters were identified according to the best consensus matrix (k = 2) **(B)** The correlations between the pyroptosis-related DEGs and clinicopathologic characters of the two clusters were shown as a heatmap **(C)** The overall survival between the two clusters.

The 24 DEGs were next applied to stratify TCGA HCC patients into different subtypes by performing consensus clustering analysis. When the clustering variable (kappa) was defined as 2, the TCGA HCC patients were then divided into two subtypes (Figure 2A). The correlations between gene expressions and clinical features such as tumor stage, tumor grade, gender and survival status were shown in a heatmap. No significant differences were found between the two groups (Figure 2B). Interestingly, there was a tendency for better overall survival time in cluster one group, but the difference was not statistically significant (Figure 2C).

### Construction of a Prognostic Model in the TCGA Cohort

**FIGURE 3:**
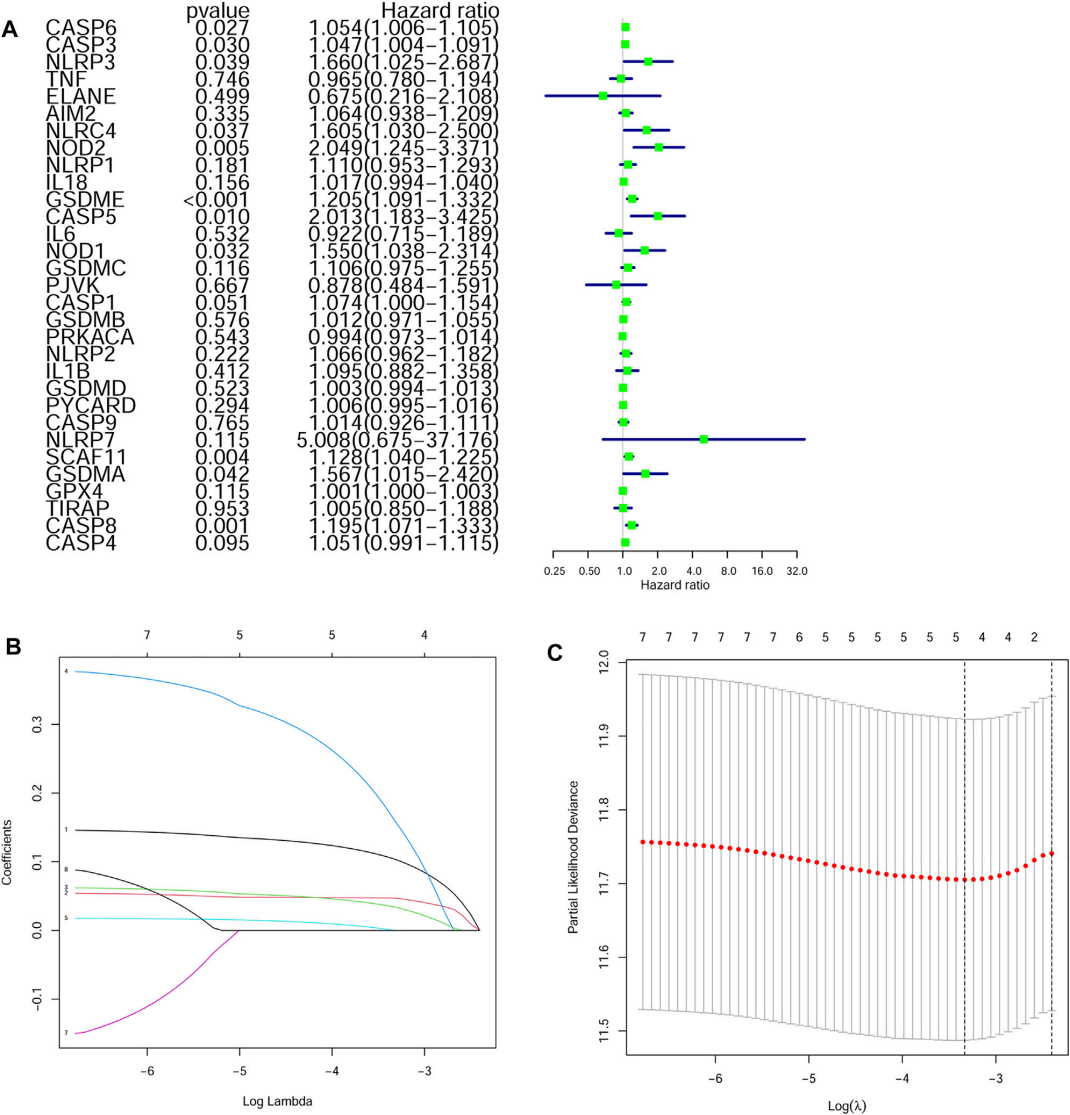
Construction of a LASSO regression model **(A)** Univariate cox regression analysis identified the prognostic related genes **(B)** LASSO regression of the eight prognostic genes **(C)** Cross-validation of the LASSO regression.

**FIGURE 4:**
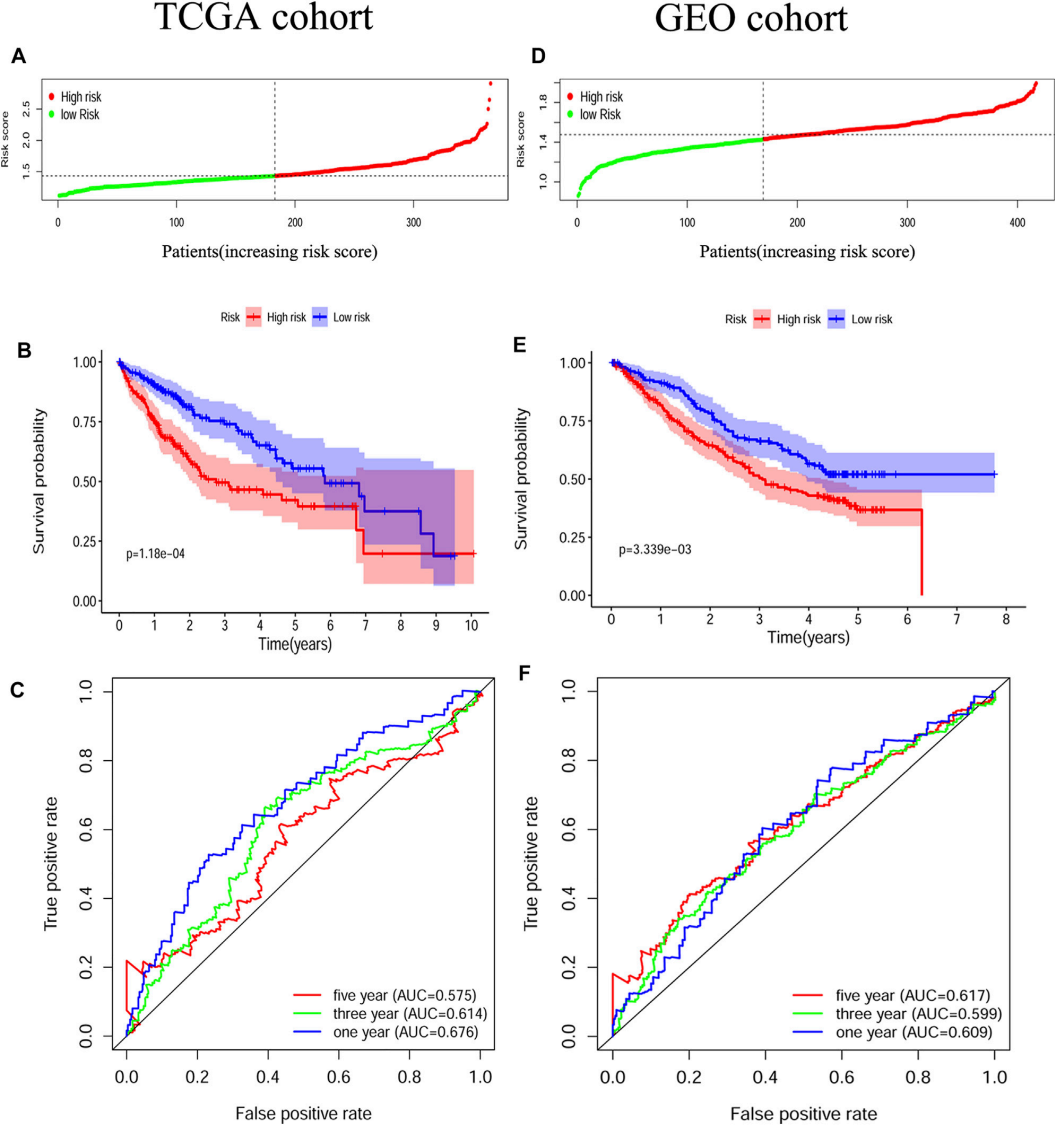
Construction and validation of the pyroptosis-related gene signature **(A)** Patients divided by the median risk score in the TCGA cohort **(B)** Survival analysis between the high-risk and low-risk groups in the TCGA cohort **(C)** ROC curves indicated the accuracy of the prognostic model in the TCGA cohort **(D)** Patients divided by the median risk score in the GEO cohort **(E)** Survival analysis between the high-risk and low-risk groups in the GEO cohort **(F)** ROC curves indicated the accuracy of the prognostic model in the GEO cohort.

The survival-related genes were selected by the univariate Cox regression analysis from the 24 DEGs (Figure 3A). Eight genes (GSDME, CASP8, SCAF11, NOD2, CASP6, CASP3, NOD1 and NLRP3) were further analyzed by LASSO-Cox regression analysis. Finally, a 5-gene signature was constructed according to the optimum *λ* value. The risk score was defined as follows: risk score= (0.1042*GSDME exp.) + (0.0471*CASP8 exp.) + (0.0344*SCAF11 exp.) + (0.1619*NOD2 exp.) + (0.0003*CASP6 exp.) (Figure 3 B/C). According to the median cut-off value, the TCGA cohort was divided into the low-risk and high-risk groups (Figure 4A). Survival analysis indicated that the low-risk group had better overall survival (*p* < 0.001, Figure 4B). Time-dependent receiver operating characteristic (ROC) curves were adopted for testing the accuracy of the prognostic model, and the area under the curve (AUC) was 0.676 for 1 year, 0.614 for 3 years, and 0.575 for 5 years (Figure 4C).

### Validation of the Prognostic Model in the GEO Cohort

HCC patients from three GEO datasets were applied for the validation of the risk model. The gene expression data were normalized as previously described before analysis. The baseline characteristics of the patients finally included were shown in Supplementary Table S3. According to the median risk score from the TCGA cohort, patients from the GEO cohort were also divided into the low-risk and high-risk groups (Figure 4D). Similar to the TCGA cohort, Kaplan–Meier analysis indicated that the low-risk group had better overall survival than the high-risk group (*p* < 0.01, Figure 4E). The AUC was 0.609 for 1 year, 0.599 for 3 years, and 0.617 for 5 years (Figure 4F).

### Independent Prognostic Value of the Gene Signature

**FIGURE 5:**
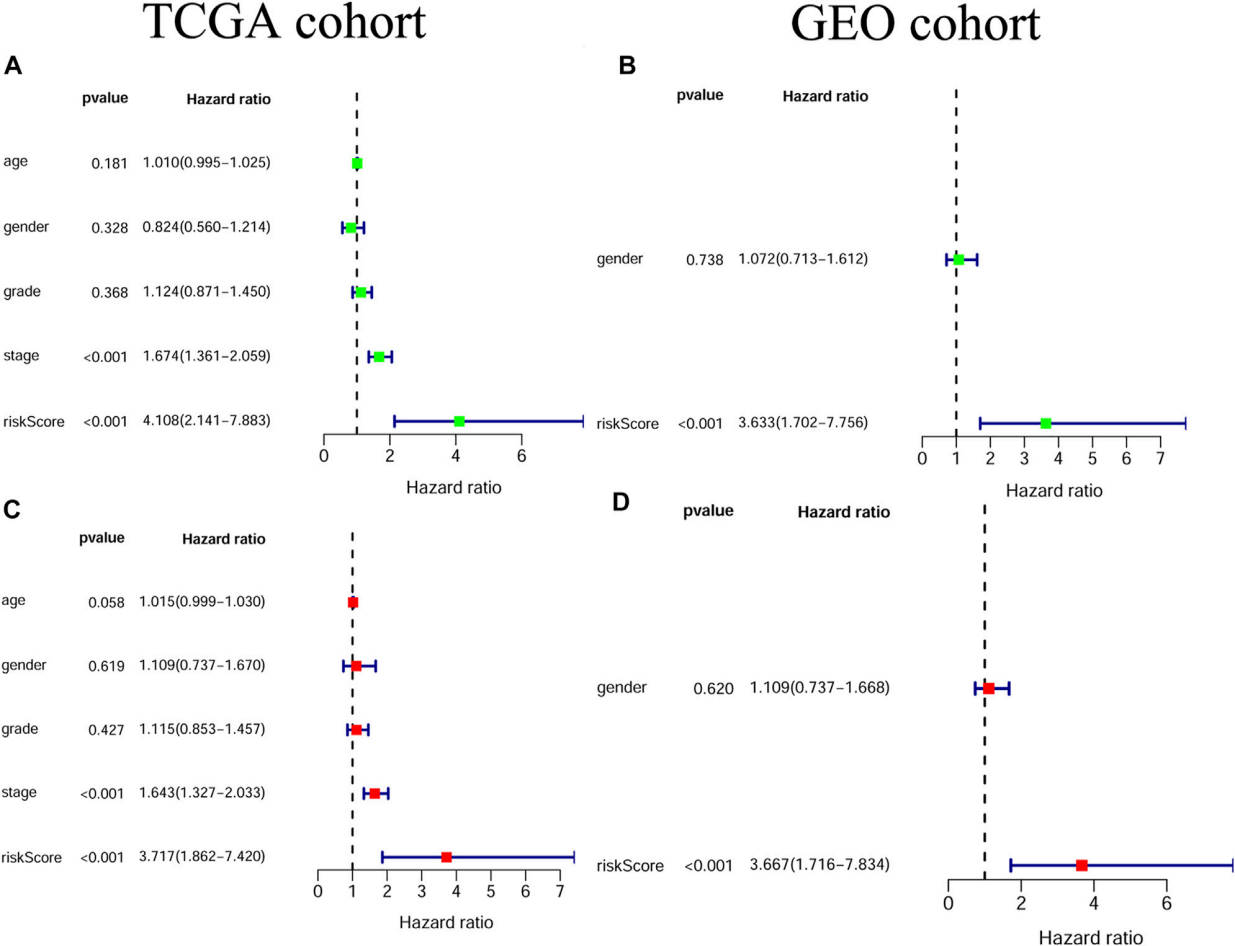
The independent prognostic value of the risk score **(A)** Univariate cox regression analysis for the TCGA cohort **(B)** Univariate cox regression analysis for the GEO cohort **(C)** Multivariate cox regression analysis for the TCGA cohort **(D)** Multivariate cox regression analysis for the GEO cohort.

Univariate and multivariable Cox regression analyses were adopted for evaluating the independent prognostic value of the risk score derived from the gene signature.The univariate Cox regression analysis showed that the risk score was a risk factor for OS in both the TCGA and GEO cohorts (TCGA cohort: HR = 4.108, 95% CI = 2.141–7.883, *p* < 0.001; GEO cohort: HR = 3.633, 95% CI = 1.702–7.756, *p* < 0.001; Figure 5 A/B). By multivariable Cox regression analysis, our results showed that the risk score was an independent prognostic factor for OS in both the TCGA and GEO cohorts (TCGA cohort: HR = 3.717, 95% CI = 1.862–7.420, *p* < 0.001; GEO cohort: HR = 3.667, 95% CI = 1.716–7.834, *p* < 0.001; Figure 5 C/D). Moreover, the results of the proportional hazard assumption test showed that our model fitted well (For TCGA model, *p* value = 0.3054, chi2 = 6.01; For GEO model, *p* value = 0.4658, chi2 = 1.53).

### Functional Enrichment Analysis Based on the Risk Score

**FIGURE 6:**
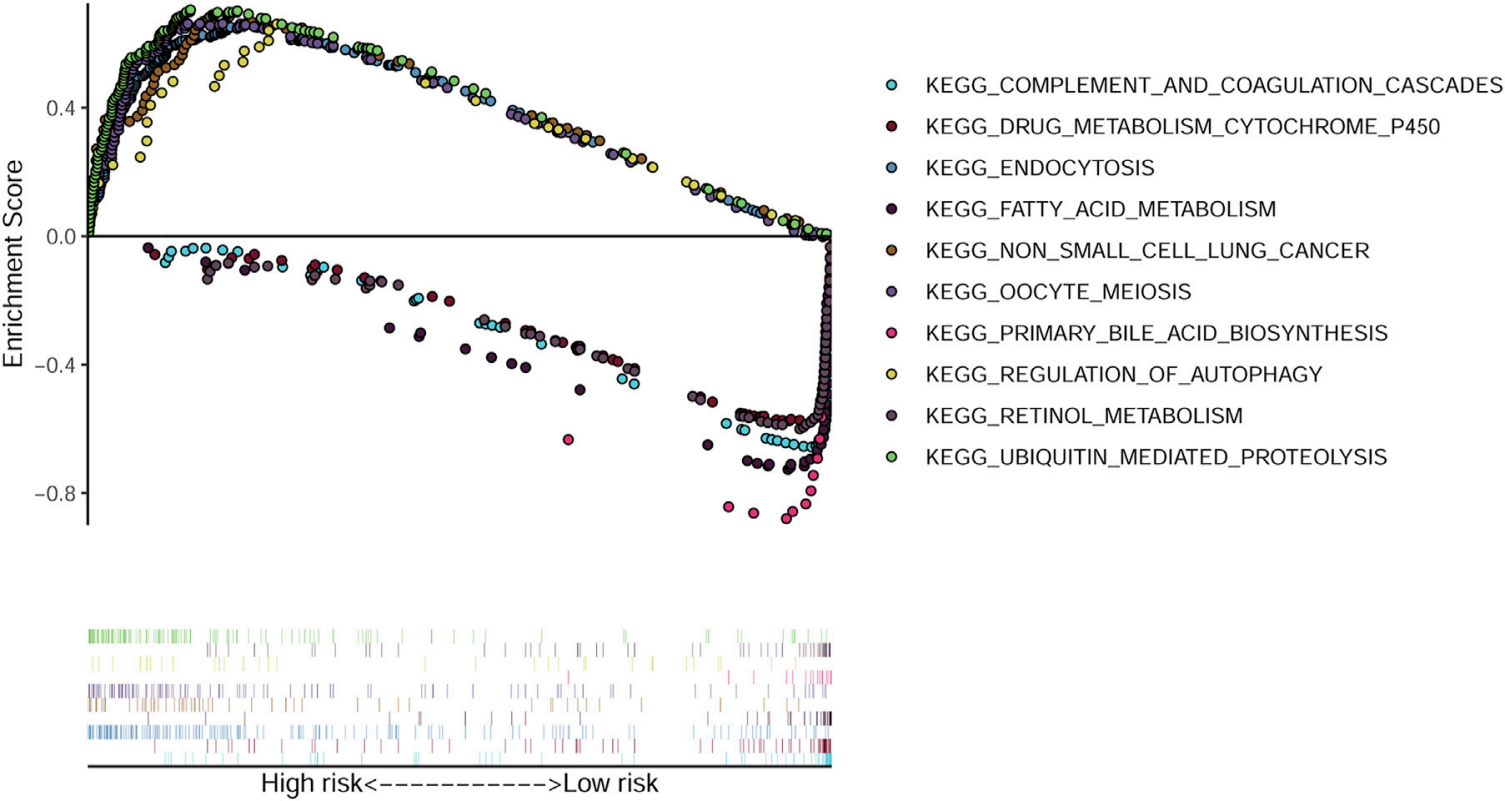
GSEA analysis identified enriched KEGG pathways. The top five enriched KEGG pathways in the high-risk and low-risk groups.

GSEA was conducted by using the TCGA data and 50 most significantly enriched KEGG pathways were identified. The endocytosis, ubiquitin mediated proteolysis and regulation of autophagy pathways were enriched in the high-risk group. While drug metabolism cytochrome P450, fatty acid metabolism and tryptophan metabolism pathways were enriched in the low-risk group (Figure 6).

### Immune Status and Prediction of Response to ICIs

**FIGURE 7:**
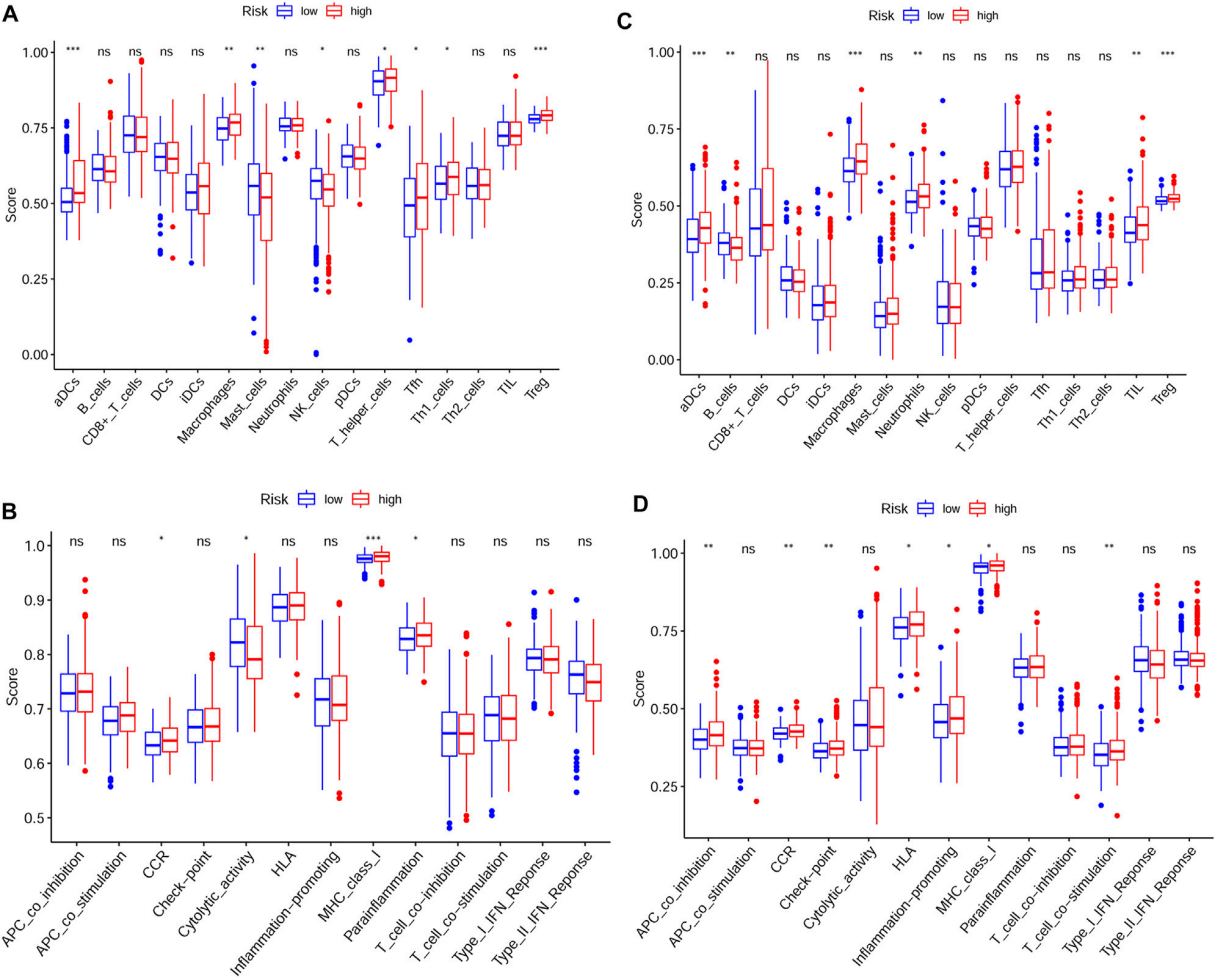
Immune status between the high-risk and low-risk groups analyzed by ssGSEA **(A,B)** Immune cells and immune-related pathways between the high-risk and low-risk groups in the TCGA cohort **(C,D)** Immune cells and immune-related pathways between the high-risk and low-risk groups in the GEO cohort (*p* values were represented as: ns not significant; *p < 0.05; **p < 0.01; ***p < 0.001.)

**FIGURE 8:**
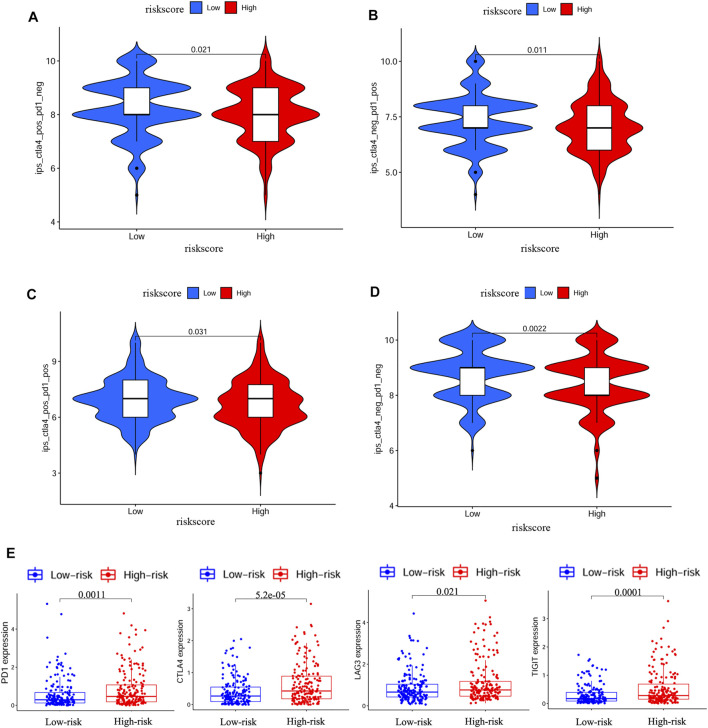
IPS analysis and correlations between risk score and common immune checkpoints **(A–D)** Comparison of the scores of IPS-CTLA4 blocker, IPS-PD1 blocker, IPS-PD1/CTLA4 blocker and IPS between different risk groups **(E)** Correlations between risk score and PD1, CTLA4, LAG3 and TIGIT.

We further explored the association between risk score and immune status. By ssGSEA, we analyzed the enrichment scores of different immune cells and immune-related pathways. As shown in Figures 7A–D, the levels of infiltration of aDCs, macrophages and Treg were elevated in the high-risk group both in the TCGA and GEO cohorts, while the CCR pathway and MHC class I pathway were also activated in the high-risk group both in the TCGA and GEO cohorts. IPS has been regarded as a well predictor for response of immunotherapy in prior studies (Charoentong et al., 2017; Yang et al., 2019). Here, we evaluated the scores of IPS, IPS-PD1 blocker, IPS-PD1/CTLA4 blocker and IPS-CTLA4 blocker. As shown in Figure 8 A/B/C/D, the scores of the above four groups were all elevated in the low-risk group, indicating that the low-risk patients may have a better response to ICIs. Besides, the correlations between risk score and common immune checkpoints including PD1, CTLA-4, LAG-3 and TIGHT were also calculated. The results showed that the expression of PD1, CTLA-4, LAG-3 and TIGHT were elevated in the high-risk group (Figure 8E). These results also indicated that the low-risk patients may have a better response to ICIs.

## Discussion

Pyroptosis is a novel kind of cellular necrosis, characterized by releasing huge amount of inflammatory factors and the formation of bubble-like morphology. It has been suggested to have both tumor protecting and promoting roles. More recently, researchers have focused on the induction of pyroptosis in tumor cells, which may be a novel therapeutic target (Xia et al., 2019). The pyroptosis-related genes have been proved to be prognostic in both ovarian cancer and lung cancer. However, the diverse roles of pyroptosis-related genes in HCC have yet to be analyzed.

For the first time, we explored the expression and prognostic roles of pyroptosis-related genes in HCC. Our results indicated that 24 genes were differentially expressed between the HCC tissues and normal tissues. A gene signature containing five pyroptosis-related genes (GSDME, CASP8, SCAF11, NOD2, CASP6) could well predict OS in HCC patients. The risk score generated from the gene signature was shown to be an independent risk factor for poor prognosis. Similar to our results, prior studies have also proved the prognostic roles of pyroptosis-related genes in lung cancer and ovarian cancer (Lin et al., 2021; Ye et al., 2021).

GSDME, also known as deafness autosomal dominant 5 (DFNA5), one of the genes from our gene signature, was first identified for its involvement in inherited hearing impairment (Van Laer et al., 1998). GSDME was one of the core executors in cell pyroptosis. GSDME was cleaved by caspase-3 into pore-formation of GSDME-N domain, which triggered pyroptosis in cells (Rogers et al., 2017). GSDME was originally identified as a tumor suppressor because its expression was downregulated in breast cancer and reduced GSDME was associated with poor survival of breast cancer patients (Kim et al., 2008). However, GSDME was also found to be overexpressed in head and neck squamous cell carcinoma, lung squamous cell carcinoma and cholangiocarcinoma. Overexpression of GSDME was associated with poor survival of head and neck squamous cell carcinoma (Liu et al., 2021; Zhang et al., 2021). Hence, GSDME may play different roles in different types of cancer. In this study, our result showed that GSDME may play a tumor promoting role in HCC. Though, small molecule drugs such as cannabidiol and miltirone may induce HCC cell death through GSDME-mediated pyroptosis (Zhang et al., 2020b; Shangguan et al., 2021), non-cleavable or pore-defective GSDME was also proved to be not tumor suppressing (Zhang et al., 2020a). The detailed role of GSDME in HCC are worthy of further exploring.

NOD2, another gene from our gene signature, is reported to promote inflammation and apoptosis. It has been indicated that NOD2 can contribute to myocardial ischemia/reperfusion injury through inducing cardiomyocyte apoptosis and inflammation (Liu et al., 2016). Emerging evidence has been found between NOD2 and human cancers. NOD2 was found to be prognostic in kidney cancer (Xu et al., 2017). NOD2 was also proved to be upregulated and activated in HCC samples, overexpression of NOD2 correlated with poor survival. Mechanically, NOD2 increased liver inflammation via inducing the nuclear autophagy pathway (Zhou et al., 2021). Similar to their results, our results showed that NOD2 was elevated in HCC tissues and correlated with poor survival.

By GSEA analysis, our results indicated that endocytosis, ubiquitin mediated proteolysis and regulation of autophagy were enriched in the high-risk group, which indicated stronger protein metabolism in the high-risk group. We speculated the hypermetabolic behavior in the high-risk group may lead to high malignancy and poor prognosis, which needed to be elucidated in further studies. In order to explore the correlations between risk score and immune cell infiltration in tumor, ssGSEA was adopted. Generally speaking, the high abundance of Treg contributes to immunosuppression and poor survival (Zhou et al., 2016; Zhang et al., 2019). What’s more, macrophages, especially the tumor associated macrophages are mainly M2 macrophages, which are shown to be tumor promoting (Deng et al., 2021). Here, in this study, our results showed that macrophages and Treg were enriched in the high-risk group and patients in the high-risk group also had poor prognosis. Although, our ssGSEA results seemed not always to be consistent, it was acceptable from an overall perspective.

Immunotherapy of ICIs has become an inspiring treatment for various of advanced cancers including HCC. Recently, results from the study of KEYNOTE-240 also proved the value of pembrolizumab as second-line therapy in patients with advanced HCC(Finn et al., 2020). However, not all HCC patients can benefit from the ICIs therapy. It is worthy selecting patients who may benefit from immunotherapy. In this study, we explored the relationship between IPS and risk score. The results suggested that the low-risk group had higher IPS scores, IPS-PD1 blocker scores, IPS-PD1/CTLA4 blocker scores and IPS-CTLA4 blocker scores, indicating the possibility of applying the risk score to predict response of immunotherapy. Besides, we also investigated the relationship between risk score and common immune checkpoints including PD1, CTLA-4, LAG-3 and TIGHT. Our results showed that the expressions of PD1, CTLA-4, LAG-3 and TIGHT were elevated in the high-risk group which also indicated that the low-risk patients may have a better response to ICIs. Thus, our predictive model may be helpful for selecting patients in clinical practice.

Nevertheless, our study had some limitations. As all the data included for analysis were retrieved from online databases, further *in vivo*/vitro studies are still needed to verify these findings. Besides, we did not explore the post-translational modifications of these genes, as post-translational modifications were of great importance for controlling intracellular signal transduction and cell functions. What’s more, the detailed signal pathways for these genes affecting clinical survival deserved to be investigated in future studies. Last but not least, with the development of single-cell RNA-seq (scRNA-seq) technique, we are now able to focusing on the specific types of cells which mainly express the pyroptosis-related genes (Zhao et al., 2020; Song et al., 2021). This would be certainly more meaningful for us to understand the HCC microenvironment. Till now, few single-cell studies have explored the role of pyroptosis in HCC, it is a promising research direction for our future study.

In conclusion, we systemically explored the expression, predictive signaling pathways, prognosis and associations with immune infiltration of pyroptosis-related genes in patients with HCC. Our research gives new insights regarding the roles of pyroptosis-related genes to HCC and may be helpful for selecting patients in clinical practice.

## Data Availability

The datasets presented in this study can be found in online repositories. The names of the repository/repositories and accession number(s) can be found in the article/Supplementary Material.
